# Efficacy of programmed intermittent bolus epidural analgesia in thoracic surgery: a randomized controlled trial

**DOI:** 10.1186/s12871-019-0780-0

**Published:** 2019-06-15

**Authors:** M. Higashi, K. Shigematsu, E. Nakamori, S. Sakurai, K. Yamaura

**Affiliations:** 1 Department of Anesthesiology, Fukuoka University School of Medicine, 7-45-1, Nanakuma, Jonan-ku, Fukuoka, 814-0180 Japan; 2 Operation rooms, Fukuoka University Hospital, Fukuoka, Japan

**Keywords:** Anesthesia, Epidural anesthesia, Programmed intermittent bolus, Thoracic surgery

## Abstract

**Background:**

Continuous epidural infusion (CEI) has some disadvantages, such as increased local anesthetic consumption and limited area of anesthetic distribution. Programmed intermittent bolus (PIB) is a technique of epidural anesthesia in which boluses of local anesthetic are automatically injected into the epidural space. The usefulness of PIB in thoracic surgery remains unclear. In this study, we aimed to compare the efficacies of PIB epidural analgesia and CEI in patients undergoing thoracic surgery.

**Methods:**

This randomized prospective study was approved by the Institutional Review Board. The study included 42 patients, who were divided into CEI (*n* = 21) and PIB groups (n = 21). In the CEI group, patients received continuous infusion of the local anesthetic at a rate of 5.1 mL/90 min. In the PIB group, a pump delivered the local anesthetic at a dose of 5.1 mL every 90 min. The primary endpoints were the frequency of patient-controlled analgesia (PCA) and the total dose of local anesthetic until 36 h following surgery. Student’s *t*-test, the chi-square test, and the Mann–Whitney *U* test were used for statistical analyses.

**Results:**

The mean number of PCA administrations and total amount of local anesthetic were not significantly different between the two groups up to 24 h following surgery. However, the mean number of PCA administrations and total amount of local anesthetic at 24–36 h after surgery were significantly lower in the PIB group than in the CEI group (median [lower–upper quartiles]: 0 [0–2.5] vs. 2 [0.5–5], *P* = 0.018 and 41 [41–48.5] vs. 47 [43–56], *P* = 0.035, respectively). Hypotension was significantly more frequent in the PIB group than in the CEI group at 0–12 h and 12–24 h (3.3% vs. 0.5%, *P* = 0.018 and 7.9% vs. 0%, *P* = 0.017, respectively).

**Conclusion:**

PIB can reduce local anesthetic consumption in thoracic surgery. However, it might result in adverse events, such as hypotension.

**Trial registration:**

This randomized prospective study was approved by the Institutional Review Board (IRB No. 15-9-06) of the Fukuoka University Hospital, Fukuoka, Japan, and was registered in the clinical trials database UMIN (ID 000019904) on 24 November 2015. Written informed consent was obtained from all patients.

## Background

Continuous epidural infusion (CEI) of a local anesthetic combined with patient-controlled analgesia (PCA) is an effective postoperative analgesic approach for thoracic surgery [1]. However, CEI has some disadvantages, such as increased local anesthetic consumption and a limited area of anesthetic distribution [2].

Programmed intermittent bolus (PIB) is a technique of epidural anesthesia in which boluses of local anesthetic are automatically injected into the epidural space. This technique increases the analgesic area [3]. Reports have indicated that intermittent epidural bolus administration reduces local anesthetic usage and improves maternal satisfaction in labor analgesia [4–6]. However, the usefulness of PIB in thoracic surgery is unclear.

The purpose of this study was to compare the efficacies of PIB epidural analgesia and CEI in patients undergoing thoracic surgery.

## Methods

This randomized prospective study was approved by the Institutional Review Board (IRB No. 15–9-06) of Fukuoka University Hospital, Fukuoka, Japan, and was registered in the clinical trials database UMIN (ID 000019904) on 24 November 2015. Written informed consent was obtained from all patients.

### Patients

**Fig. 1:**
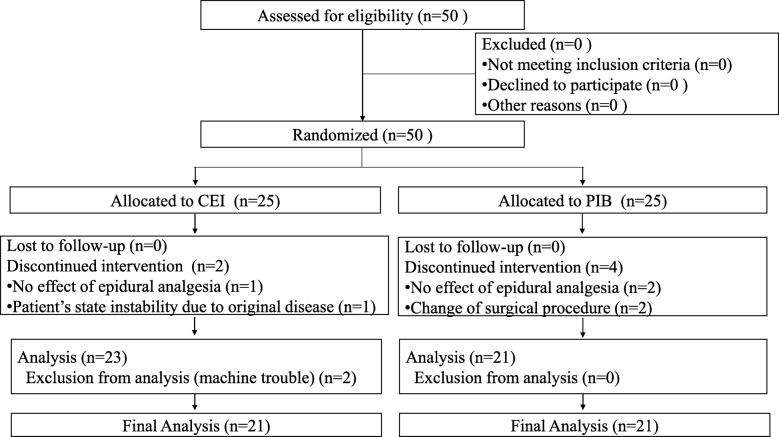
Flow chart of this study

Patients undergoing open lung lobectomy or partial lobectomy at the Fukuoka University Hospital, Fukuoka, Japan between March 2016 and March 2017 were recruited. The exclusion criteria were age < 20 years and contraindication for epidural anesthesia. Patients were randomly divided into a CEI or PIB group by computer generated randomization using Excel 2013 (Microsoft Inc., Redmond, WA) by KY (Fig. 1).

The study was discontinued when epidural analgesia was ineffective, when the patient’s hypotension continued even after the administration of vasopressor, or when motor paralysis appeared owing to epidural analgesia.

### Anesthesia

Under standard monitoring, thoracic epidural anesthesia was performed at Th4–6 in the lateral position. An 18G epidural Tuohy needle (Uniever®, Unisis Corp., Saitama, Japan) was used, and the epidural space was identified using the loss-of-resistance technique. A 20G epidural catheter (Uniever®, Unisis Corp.) was inserted 5 cm to the head side. Following a 3-mL test dose of 1% mepivacaine, the epidural catheter was fixed.

General anesthesia was induced with intravenous fentanyl (2 μg/kg), propofol (1 mg/kg), and rocuronium (0.9 mg/kg) and was maintained with sevoflurane (1.5–2%) and remifentanil (0.1–0.2 μg/kg/min). Fentanyl was used intravenously up to 5 μg/kg. A local anesthetic via the epidural catheter was not used during the operation.

After surgery, all patients were extubated in the operating room, observed in the post-anesthesia care unit for 30 min to 1 h, and then transferred to the ward.

### Intervention

At the end of surgery, a 5-mL initial dose of local anesthetic (ropivacaine 2 mg and fentanyl 2 μg in 1 ml) was administered via the epidural catheter after closure of thoracotomy in both groups.

**Fig. 2:**
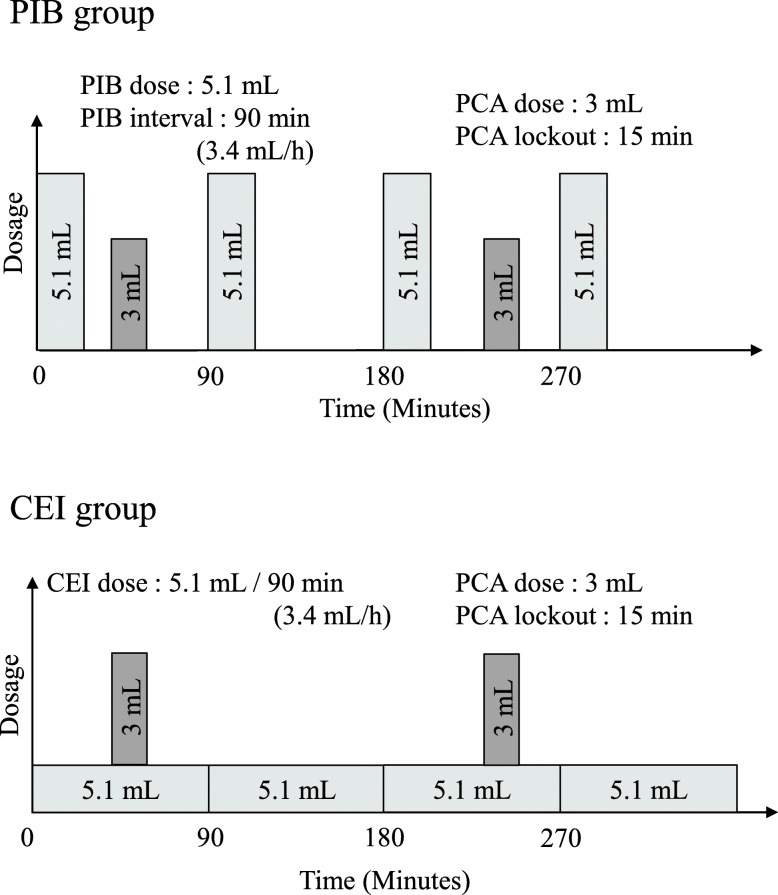
Study protocol. PIB: programmed intermittent bolus, PCA: patient controlled analgesia, CEI:continuous epidural infusion

The study protocol is shown in Fig. 2. In both the PIB and CEI groups, a pump (CADD-Solis ambulatory infusion pump, Smith Medical, St Paul, MN, USA) was used. In the CEI group, patients received continuous infusion of the local anesthetic at a rate of 5.1 mL/90 min (3.4 mL/h). In the PIB group, the pump delivered the local anesthetic at a dose of 5.1 mL every 90 min. The PCA system was programmed to deliver a 3-mL bolus of the local anesthetic with a lockout interval of 15 min in both groups.

The primary endpoints were the frequency of PCA and total dose of local anesthetic during 36 h of postoperative period. The secondary endpoints were pain intensity, frequency of rescue analgesics, including nonsteroidal anti-inflammatory drugs and acetaminophen, adverse events, hypotension, and postoperative nausea and vomiting (PONV). Hypotension was defined as systolic blood pressure (SBP) 20% less than the baseline value or less than 90 mmHg. The onset of adverse reactions and use of rescue analgesics postoperatively were examined. The pain intensity was assessed using a visual analog scale (VAS) during rest, deep breathing, cough, and movement.

### Statistical analysis

Continuous variables are expressed as mean ± standard deviation or median [lower–upper quartiles]. From the results of preliminary study, total dose of local anesthetics in CEI was 24 ml more than in PIB, and SD of CEI was 32. Based on these results, we estimated that the following: SD = 32, Δ = 0.78, α = 0.05, and beta = 0.2. The required number of cases was estimated to be 21 for each group. We considered a 10–20% dropout rate; therefore, 50 patients were enrolled. Differences between groups were examined for statistical significance by using student’s *t*-test after logarithmic transformation. Student’s *t*-test, the chi-square test, and the Mann–Whitney *U* test were used for statistical analyses. A *P*-value < 0.05 was considered statistically significant.

## Results

Fifty patients who underwent open lung lobectomy or partial lobectomy were randomly divided into the CEI group (*n* = 25) and PIB group (n = 25). In the CEI group, 2 patients were excluded because of ineffectiveness of epidural analgesia and instability in the patient’s state due to the original disease. In the remaining 23 patients of the CEI group, additional 2 patients were excluded from the analysis because of machine trouble; finally, 21 patients were included in the analysis. In PIB group, 4 patients were excluded (2 patients owing to ineffectiveness of epidural analgesia and 2 patients owing to change in surgical procedure), and 21 patients were finally included in the analysis (Fig. 1). Patient characteristics are shown in Table 1.

**Table 1:** Patient characteristics

	PIB (*n* = 21) mean ± SD	CEI (*n* = 21) mean ± SD	*P*-value
Age (years)	63 ± 2.8	67 ± 2.6	0.34
BMI (kg/m^2^)	24.5 ± 1.1	22.4 ± 0.5	0.08
SBP (mmHg)	122 ± 2	121 ± 2	0.72
Use of analgesics during the operation			
Fentanyl (μg)	171 ± 35	193 ± 35	0.67
Remifentanil (mg)	1.5 ± 0.2	1.8 ± 0.2	0.32
Operation time (min)	249 ± 20	263 ± 18	0.61
Anesthesia time (min)	334 ± 22	351 ± 18	0.56

**Fig. 3:**
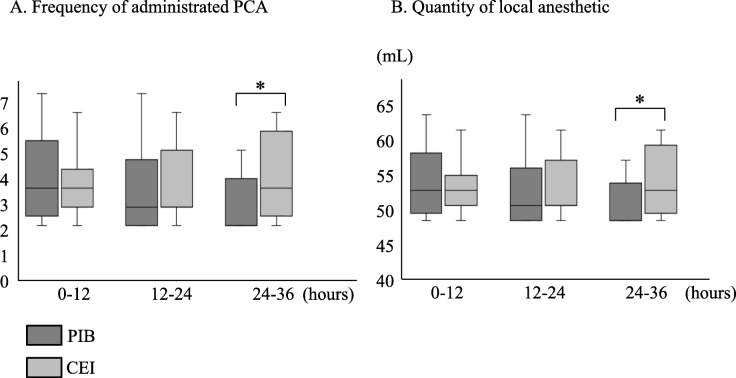
PCA data after surgery. Data are presented as median [lower–upper quartiles]. The Mann–Whitney *U* test was used for comparison of categorical variables. *P < 0.05. PCA: patient-controlled analgesia, PIB: programmed intermittent bolus, CEI: continuous epidural infusion

**Fig. 4:**
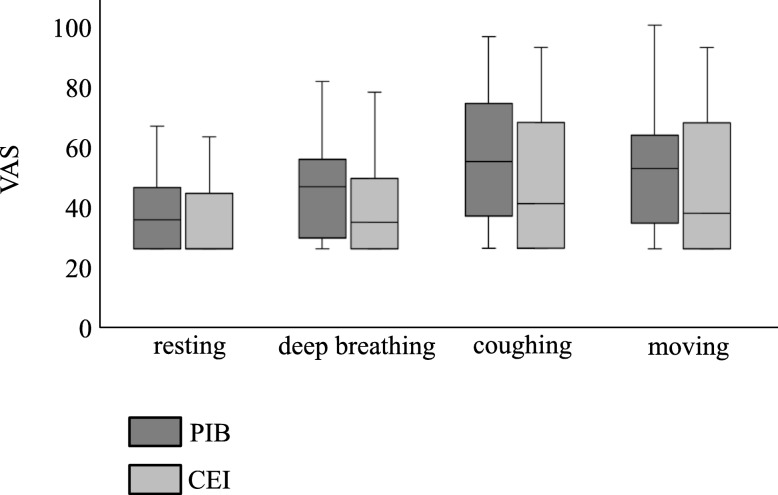
VAS scores at POD 1. Data are presented as median [lower–upper quartiles]. The Mann–Whitney *U* test was used for comparison of categorical variables. VAS: visual analog scale, PIB: programmed intermittent bolus, CEI: continuous epidural infusion, POD: postoperative day

The mean number of PCA administrations and total amount of local anesthetic were not significantly different between the two study groups up to 24 h after surgery. However, the mean number of PCA administrations was significantly lower in the PIB group than in the CEI group at 24–36 h after surgery (median [lower–upper quartiles]: 0 [0–2.5] vs. 2 [0.5–5], *P* = 0.018) and total amount of local anesthetic was also significantly lower in the PIB group than in the CEI group at 24–36 h after surgery (median [lower–upper quartiles]: 41 [41–48.5] vs. 47 [43–56] mL, *P* = 0.035) (Fig. 3). The use of rescue analgesics was not significantly different between the two study groups (Table 2). The VAS scores during resting, deep breathing, coughing, and moving after the surgery were also not significantly different between the two study groups (Fig. 4).

**Table 2:** Use of rescue analgesics postoperatively

	PIB (*n* = 21)	CEI (*n* = 21)	*P*-value
	n (%)	n (%)	
Use of analgesics			
Loxoprofen	20(95.2)	19(85.6)	0.55
Acetaminophen	1(4.7)	3(14.3)	0.61
Celecoxib	1(4.7)	2(9.5)	0.55
Tramadol	2(9.5)	1 (4.7)	0.55

The frequencies of adverse events, such as nausea, vomiting, and dizziness on standing up, were not significantly different between the two study groups (Table 3). The frequency of hypotension was greater in the PIB group than in the CEI group at 0–12 h and 12–24 h postoperatively (3.3% vs. 0.5%, *P* = 0.018 and 7.9% vs. 0%, *P* = 0.017, respectively) (Table 4).

**Table 3:** Adverse events

	PIB (*n* = 21)	CEI (*n* = 21)	*P*-value
	n (%)	n (%)	
Adverse effects			
Nausea, vomiting	8(38.1)	5(23.8)	0.32
Urinary retention	2(9.5)	3(14.3)	0.63
Feeling dizzy on standing up	4(19.0)	1(4.7)	0.15

**Table 4:** Frequency of hypotension events after surgery

	PIB (*n* = 21)	CEI (*n* = 21)	*P*-value
	n (%)	n (%)	
20% less than baseline SBP			
0-12 h	7(3.3)	1(0.5)	0.018
12-24 h	6(9.5)	3(4.7)	0.26
24-36 h	6(9.5)	4(6.3)	0.47
SBP less than 90 mmHg			
0-12 h	10(4.8)	4(1.9)	0.0495
12-24 h	5(7.9)	0(0.0)	0.017
24-36 h	4(6.3)	1(1.6)	0.15

## Discussion

Our results showed that PIB has an analgesic effect comparable with that of CEI and reduces the required amount of local anesthetic on the first day after thoracotomy. However, adverse events, such as hypotension, need attention.

To compensate for the limitations of CEI, such as a restricted area of analgesic effect, the technique of intermittent bolus infusion of epidural analgesics has been developed. The advantage of PIB is mainly in the maintenance of labor analgesia [5, 7]. Its use has been recently demonstrated in total knee arthroplasty and major abdominal and gynecological surgery, and its utility has been shown [8–10]. However, to our knowledge, this is the first randomized study to show the advantage of PIB in thoracotomy.

The reduction in the total amount of local anesthetic with intermittent bolus infusion compared with continuous infusion is consistent with the findings in labor analgesia reports and postoperative reports. In major abdominal and gynecological surgery, the beneficial effect of PIB is noted on the first postoperative day and not on the day of the operation [9]. Sequential epidural bolus infusion provides superior epidural block compared with CEI [2].

Compared with bolus infusion, hemodynamic stability with CEI without bolus administration is superior; the incidence of hypotension reduced by 67% without using bolus infusion compared with that using bolus infusion [11]. However, PIB studies for postsurgical analgesia indicated no adverse effects [8–10]. With regard to the incidence of hypotension, the difference between our results and those of previous reports might be associated with differences in the site of epidural anesthesia and dose of local anesthetic. Hypotension occurred but was not significant in both groups, and there was a need for noradrenalin when epidural anesthesia involved puncture at Th8–10 [9]. On the other hand, when epidural anesthesia involved puncture at Th10–12 in open gynecological surgery [8] or L3–5 in total knee arthroplasty, [8] there was no hypotension requiring intervention. The bolus dose was 6 mL every hour in the major surgical study that reported hypotension, [9] and among studies that did not report hypotension, the doses were 4 mL every hour for open gynecological surgery [10] and 3 mL every hour for total knee arthroplasty [8]. We used a bolus of 5.1 mL every 90 min (3.4 mL every hour). Therefore, when PIB and CEI are used for a higher level of thoracic epidural anesthesia, attention should be paid to the bolus dose to avoid hypotension.

The present study has limitations. First, this is not double blinded study. Second, in this study, the dose and concentration of local anesthetic was single, and the total dose of local anesthetic and counts of PCA were less than that of preliminary studies. We need to re-examine the small dose and concentration of local anesthetics in future studies.

## Conclusions

PIB can reduce local anesthetic consumption in thoracic surgery. However, it might result in adverse events, such as hypotension.

## Data Availability

The datasets generated and/or analyzed during the current study will be available from the corresponding author on reasonable request.

## Abbreviations

BMI: Body mass index
CEI: Continuous epidural infusion
PCA: Patient-controlled analgesia
PIB: Programmed intermittent bolus
PONV: Postoperative nausea and vomiting
SBP: Systolic blood pressure
UMIN: University hospital medical information network
VAS: Visual analog scale
